# Ensiling hybrid *Pennisetum* with lactic acid bacteria or organic acids improved the fermentation quality and bacterial community

**DOI:** 10.3389/fmicb.2023.1216722

**Published:** 2023-06-29

**Authors:** Qixian Feng, Juan Zhang, Wenqing Ling, Abraham Allan Degen, Yi Zhou, Chenyan Ge, Fulin Yang, Jing Zhou

**Affiliations:** ^1^College of Animal Sciences (College of Bee Science), Fujian Agriculture and Forestry University, Fuzhou, China; ^2^Desert Animal Adaptations and Husbandry, Wyler Department of Dryland Agriculture, Blaustein Institutes for Desert Research, Ben-Gurion University of the Negev, Beer Sheva, Israel; ^3^China National Engineering Research Center of JUNCAO Technology, Fujian Agriculture and Forestry University, Fuzhou, China

**Keywords:** calcium propionate, hybrid *Pennisetum*, silage, *Lactiplantibacillus plantarum*, microbial diversity, propionic acid

## Abstract

The aim of this study was to compare the effect of different additives on nutritional quality, fermentation variables and microbial diversity of hybrid *Pennisetum* silages. A control (CK – no additives) and seven treatments were tested, namely, *Lactiplantibacillus plantarum* (LP), *Lentilactobacillus buchneri* (LB), propionic acid (PA), calcium propionate (CAP), LP + LB; LP + PA and LP + CAP. In comparison with CK, all treatments increased the contents of crude protein and lactic acid, decreased the content of butyric acid, and altered the bacterial communities of the silage. Except for the CAP and LP + CAP treatments, the additives decreased pH and the ammonia nitrogen:total nitrogen (NH_3_-N:TN) ratio. The results of principal component analysis revealed that the PA, LP + PA and LP + LB treatments ranked as the top three silages. The PA and LP + PA treatments exhibited higher water-soluble carbohydrate content, but lower pH, and NH_3_-N:TN ratio than the other treatments. With the PA and LP + PA treatments, the relative abundances of *Lactobacillus* and *Enterobacter* decreased, and of *Proteobacteria* and *Delftia* increased, while the carbohydrate metabolism of the microorganisms improved. The LP and LB treatments reduced the Shannon and Simpson diversities. In the beta diversity, PA and LP + PA separated from the other treatments, indicating that there were differences in the composition of bacterial species. The relative abundance of *Lactobacillus* increased in the LP and LB treatments and of *Leucanostoc* and *Weissella* increased in the CAP and LP + CAP treatments. In summary, the addition of *L. plantarum*, *L. buchneri*, propionic acid, calcium propionate, and their combinations improved fermentation quality, inhibited harmful bacteria and conserved the nutrients of hybrid *Pennisetum*.

## 1. Introduction

With the rapid development of the livestock industry and the increased demand for animal products, more animal feed is needed. This has increased the cost of animal feed and the competition for land between food for humans and feed for livestock (Sandström et al., 2022; Zhao et al., 2022), which has led to utilization of different forage resources for ruminants (Li D. et al., 2019; Wang N. et al., 2022). Hybrid *Pennisetum* (*Pennisetum americanum* × *Pennisetum purpureum*) has been incorporated into animal feed and has great potential to be used for ruminants. It is distributed widely in southern China, and is characterized by low cultivation input, high biomass, and strong stress tolerance (Song et al., 2019; Cai et al., 2020; Wu et al., 2020).

Ensiling is a traditional method of preserving forage (Ren et al., 2018; Drouin et al., 2021). However, the hybrid *Pennisetum* is difficult to ensile because of its low lactic acid bacteria (LAB) count (Shah et al., 2020). Bacterial inoculants and chemicals are often added to improve the fermentation and nutrient qualities of the silage, and to inhibit the activity of harmful microbiota (Queiroz et al., 2018; He Q. et al., 2021). The LAB inoculants are known for their ability to alter fermentation patterns, and are added widely to improve fermentation in the production of silage (Ogunade et al., 2017). *Lactiplantibacillus plantarum*, the most common additive, is a homofermentative LAB that produces lactic acid efficiently and reduces the pH rapidly (Xu et al., 2021; Xian et al., 2022). *Lentilactobacillus buchneri*, also a heterofermentative LAB, produces acetic acid during fermentation, inhibits yeast and mold, improves aerobic stability and reduces feed loss (Romero et al., 2017; Zhang et al., 2020). Propionic acid is an aerobic microbial inhibitor that affects nitrogen conversion and reduces the degradation of protein by acidizing the silage or limiting the activity of undesirable bacteria at the early stage of fermentation (Carvalho et al., 2012; He et al., 2020; Ren et al., 2021). Due to the volatility of propionic acid and its relatively short residual time, calcium propionate (CAP), which has the same antibacterial effect as propionic acid after ionization in water, was developed (Xiong et al., 2017). The above-mentioned additives have advantages, but little information is available on their effect on silage quality and the bacterial community of hybrid *Pennisetum*. To fill this knowledge gap, we compared the effects of *L. plantarum*, *L. buchneri*, propionic acid, calcium propionate, and their combinations on the chemical composition, fermentation quality, and microbial community of hybrid *Pennisetum*.

## 2. Materials and methods

### 2.1. Silage preparation

Hybrid *Pennisetum* was harvested in Fujian Province (117.93 °E, 26.79 °N, subtropical monsoon climate) in May 2021 by manually mowing at 8–10 cm above ground level, and was transported to the laboratory immediately. The *Pennisetum* was spread out evenly, and air-dried for 6 h, resulting in a dry matter (DM) content of 181.3 g/kg fresh weight (FW), and was chopped into 1–2 cm lengths using a paper cutter. The following were added to the *Pennisetum*: (1) distilled water (CK); (2) *L. plantarum* (LP, provided by Fujian Academy of Agricultural Sciences, viable counts ≥1 × 10^6^ cfu/g FW); (3) *L. buchneri* (LB, BNCC187961, Beijing Beina Chuanglian Biotechnology Institute, Beijing, China, viable counts ≥1 × 10^6^ cfu/g FW); (4) propionic acid (PA, 0.5% FW, analytical pure, Fuzhou Mili Biotechnology Co., Ltd., Fuzhou, China); (5) calcium propionate (CAP, 0.5% FW, Fuzhou Mili Biotechnology Co., Ltd., Fuzhou, China); (6) LP + LB; (7) LP + PA; and (8) LP + CAP. Each additive was dissolved in 10 mL sterile water and sprayed evenly onto the surface of the Pennisetum (CK was sprayed with an equal volume of distilled water). Subsequently, 400 g of the sprayed hybrid *Pennisetum* samples were placed in a polyethylene bag (248 mm × 344 mm), with 3 replicates for each treatment. The bags were vacuum sealed, and ensiled at room temperature of 26°C for 30 or 60 days.

### 2.2. Nutritional composition and fermentation variables of hybrid *Pennisetum* silage

After ensiling, DM content of the silage was determined by oven drying at 65°C for 48 h, and the oven-dried samples were sieved through a 0.425 mm screen. The content of water-soluble carbohydrates (WSC) was determined by anthrone sulfuric acid colorimetry (Fu and Diao, 2007); total nitrogen (TN) was determined using an automatic nitrogen analyzer (K9840 Kjeldahl, Hanon, Jinan, China), the crude protein (CP) content was calculated as TN × 6.25; and the neutral detergent fiber (NDF) and acid detergent fiber (ADF) contents were measured following Van Soest et al. (1991).

Ten g of sample were added to 90 mL of distilled water for 24 h at 4°C, and filtered through 4 layers of gauze for pH determination (pHS-3D, Shandong, China). Ammonia nitrogen (NH_3_-N) was determined by phenol sodium hypochlorite colorimetry (Arthur Thomas, 1977), and lactic, acetic, propionic, and butyric acids were determined using high-performance liquid chromatography (Wang et al., 2020).

### 2.3. The pH and microbial count during aerobic exposure

The pH and microbial counts of the treatments after 60 days of ensiling were determined at 0, 3, 6, and 9 days after aerobic exposure. The pH was determined as described previously. The methods of Dong et al. (2017) were used to measure the counts of LAB, yeast, and aerobic bacteria during aerobic exposure with the de Man Rogosa Sharpe medium, Potato Dextrose Agar medium, and Plate Count Agar medium, respectively (Fuzhou Mili Biotechnology Co., Ltd., Fuzhou, China). No antibiotics were added to the culture media.

### 2.4. Microbial diversity analysis

After 60 days of ensiling, a sample of each treatment was stored at −80°C for microbial diversity determination. High throughput sequencing was performed in triplicate, and total DNA was extracted using the CTAB/SDS method to check DNA concentration and purity with a 1% agarose gel. The 16S rDNA gene of the bacterial V3 ~ V4 hypervariable region was obtained by primer sequences 338F (ACTCCTACGGGAGGCAGCAG) and 806R (GGACTAC HVGGGTWTCTAAT) (Ling et al., 2022). PCR amplicons were identified by agarose gel electrophoresis using NEXTFLEX^®^ Rapid DNA-Seq Kit for Miseq library construction and sequencing. After the library qualified, it was sent to Majorbio BioPharm Technology Co. Ltd. (Shanghai, China) for sequencing. The library was paired-end sequenced based on the Illumina Novaseq sequencing platform resulting in a complete microbial community.

### 2.5. 16S rRNA gene sequence analysis

Raw fastq files were demultiplexed and quality filtered using Trimomatic, and further merged by FLASH software. Uparse software (Uparse v7.0.1001)^1^ was used to cluster the entire high-quality sequences of all samples, and, by default, the sequences were clustered to operational taxonomic units (OTUs) with 97% similarity (He L. et al., 2021). Alpha diversity was determined using species richness indices (Ace and Chao 1) and species diversity indices (Shannon and Simpson) (Zheng et al., 2020). Beta diversity was determined using principal coordinates analysis (PCoA), and was further analyzed using the ANOSIM test (Dong et al., 2017). The metabolic function of bacteria was predicted by comparing the Kyoto Encyclopedia of Genes and Genomes (KEGG) database with the Phylogenetic Investigation of Communities by Reconstruction of Unobserved States 2 (PICRUSt2) (Langille et al., 2013). Spearman’s correlation tested the relationships between silage quality and the relative abundances of bacteria at the genus levels in each treatment. Raw sequencing files and associated metadata have been deposited in NCBI’s Sequence Read Archive (PRJNA946341).

### 2.6. Statistical analyses

The data of silage quality were analyzed by the GLM program in SPSS software (version 26.0, Chicago, IL, United States) with ensiling time, additive treatment and their interaction. The principal component analysis used SPSS software. The eigenvalues of the variance matrix, the variance contribution rate and the weight coefficient of each factor were calculated to generate the principal component equation. The principal component comprehensive score was calculated by standardizing the original data into the principal component equation (Tharangani et al., 2021).

## 3. Results and discussion

### 3.1. Nutritional quality of hybrid *Pennisetum* silage

The DM and WSC contents of hybrid *Pennisetum* silage were affected by ensiling time, additives and their interactions (Table 1). When compared to CK, the LP treatment had a lesser (*p* < 0.05) DM content, while the LP + LB, PA, LP + PA, CAP and LP + CAP treatments had greater (*p* < 0.05) DM contents. The loss of DM from silage was due to the breakdown of nutrients, as aerobic microbes converted carbohydrates to water, carbon dioxide, and heat (Haas et al., 2011). In the present study, the DM content in the PA treatment was greater than in the other treatments after 60 days of ensiling. It is likely that the addition of propionic acid inhibited the growth of undesirable microorganisms and reduced their consumption of nutrients (Li et al., 2022). Propionic acid has antifungal properties by maintaining its activity on the surface of microorganisms and competing with amino acids for enzyme activity sites or by altering the cell permeability of the organisms (Gheller et al., 2021).

**Table 1:** Nutritional quality of hybrid *Pennisetum* silage after 30 and 60 days of ensiling.

Ensiling days	Treatments								SEM	*P*		
	CK	LP	LB	LP + LB	PA	LP + PA	CAP	LP + CAP		D	T	D × T
Dry matter (g/kg FW)												
30	201.0^b^	196.5^a^	202.5^Ab^	206.5^Ac^	220.5^Ad^	265.0^Bg^	224.5^e^	248.5^Bf^	3.404	<0.001	<0.001	<0.001
60	207.0^b^	193.5^a^	206.1^Bb^	219.0^Bd^	239.5^Bf^	211.0^Ac^	224.5^e^	221.5^Ade^				
Crude protein (g/kg DM)												
30	94.6^Ba^	113.0^b^	110.1^b^	119.6^Bbc^	126.6^cd^	134.6^Bd^	123.8^Bc^	123.5^Bc^	2.008	<0.001	<0.001	0.009
60	82.5^Aa^	103.2^b^	109.3^bc^	108.7^Abc^	123.0^d^	114.4^Acd^	101.5^Ab^	100.2^Ab^				
Water-soluble carbohydrates (g/kg DM)												
30	21.6^Bb^	25.1^Bc^	24.4^Bc^	24.1^Bc^	38.8^Bd^	42.1^Be^	18.8^Ba^	20.0^Bab^	1.502	<0.001	<0.001	<0.001
60	13.7^Ae^	11.5^Ad^	10.8^Ad^	9.0^Ac^	23.8^Ag^	21.7^Af^	4.0^Ab^	6.3^Aa^				
Neutral detergent fiber (g/kg DM)												
30	633.5^Bd^	604.0^cd^	606.0^cd^	548.5^b^	582.2^bc^	504.9^a^	613.6^cd^	581.8^bc^	6.131	<0.001	0.001	0.262
60	570.5^Aab^	572.2^ab^	586.1^b^	544.3^ab^	521.9^a^	514.4^a^	560.7^ab^	557.5^ab^				
Acid detergent fiber (g/kg DM)												
30	393.6^Bb^	363.3^Bb^	388.7^Bb^	327.4^a^	362.5^Bab^	330.0^a^	383.6^Bb^	352.2^ab^	4.798	0.004	<0.001	0.058
60	342.6^Aab^	359.1^b^	343.1^Aab^	321.6^ab^	300.6^Aa^	315.1^ab^	313.4^Aab^	350.5^b^				

FW, fresh weight; DM, dry matter; CK, distilled water; LP, *L. plantarum*; LB, *L. buchneri*; LP + LB, *L. plantarum* and *L. buchneri*; PA, propionic acid; LP + PA, *L. plantarum* and propionic acid; CAP, calcium propionate; LP + CAP, *L. plantarum* and calcium propionate. D = ensilage days effect; T = treatment effect; D × T = the interaction between ensiling days and treatments. Means of treatment within a row followed by different lowercase superscripts differ from each other (*p* < 0.05). Means of ensiling time within a column followed by different uppercase superscripts differ from each other (*p* < 0.05).

With the prolongation of ensiling, the CP content in each treatment decreased. The degradation of protein during ensiling involves a series of plant and microbial enzymes (Xiong et al., 2017). Proteins are converted into free amino acids and peptides through the catalytic hydrolysis of plant enzymes, and then are degraded further (Zheng et al., 2022). In the present study, the CP content was greater (*p* < 0.05) with each additive than in CK. Previous studies reported that propionic acid inhibited *Clostridia* and *Enterobacteria* effectively, as these bacteria were poor acid resistant bacteria, and reduced protein breakdown (Ali and Tahir, 2021). Adding calcium propionate also reduced CP loss, but less so than propionate, which might be due to the lesser concentration of dissociated propionate ions with CAP. The increase (*p* < 0.05) of CP content with the LB treatment most likely involved propionic acid. *L. buchneri* produces 1,2-propanediol from sugars and then propionic acid in the metabolic process, resulting in a bacteriostatic effect (Ling et al., 2022).

The WSC content of all silages was lower (*p* < 0.05) at 60 days than at 30 days of ensiling. The WSC serves as an energy source for microorganisms and its consumption implies microbial activity (Gheller et al., 2021). The WSC is converted into organic acid to reduce the pH of the silage (Zhang et al., 2019; Zhu et al., 2022). The NDF content of silage was reduced (*p* < 0.05) with the LP + LB treatments. Similarly, Du et al. (2022) reported that the content of NDF in ryegrass silage inoculated with *L. plantarum*, *L. buchneri* and *L. casei* was reduced after 60 days of ensiling. When ensiled for 30 days, the PA and LP + PA treatments had a lesser (*p* < 0.05) NDF content than CK. This could be due to the increase in total organic acids after the addition of PA, which could hydrolyze digestible cell walls (Jiang et al., 2020; Ren et al., 2020).

### 3.2. Fermentation quality of hybrid *Pennisetum* silage

Table 2 presents the effect of different additives on the silage fermentation parameters of hybrid *Pennisetum*. A pH in the range of 3.6–4.2 for silage is considered optimal, as it effectively reduces undesirable microorganisms (Lv et al., 2020; Bao et al., 2023). In the current study, the pH was below 4.2 at 30 days of ensiling in all treatments except for CAP, LP + CAP and LB, in which the pHs were greater (*p* < 0.05) than for CK. Li M. et al. (2019) concluded that CAP led to an increase in buffering energy and, thus, a rise in pH in the silage, which could explain the results in the present study. *L. plantarum*, which is regarded as the most commonly used homofermentative LAB, has the ability to reduce pH rapidly (Muck et al., 2018; Bai et al., 2022). *L. buchneri* could improve the aerobic stability of silage (Magnusson and Schnürer, 2001), and when combined with *L. plantarum*, reduced the pH at the initial stage of fermentation. Consequently, the pH of the LP + LB treatment was lesser than the LP and LB treatments.

**Table 2:** Fermentation characteristics of hybrid *Pennisetum* silage after 30 and 60 days of ensiling.

Ensiling days	Treatments								SEM	*P*		
	CK	LP	LB	LP + LB	PA	LP + PA	CAP	LP + CAP		D	T	D × T
pH												
30	4.14^Ad^	4.10^Ad^	4.36^Be^	3.88^c^	3.60^Bb^	3.46^a^	4.62^Af^	4.68^Ag^	0.068	0.045	0.465	0.046
60	4.30^Bd^	4.19^Bc^	4.18^Ac^	3.87^b^	3.49^Aa^	3.49^a^	4.89^Be^	4.90^Be^				
Lactic acid (g/kg DM)												
30	8.02^Ab^	10.31^Ad^	8.86^Ac^	13.37^e^	14.03^Af^	10.13^Ad^	8.03^Ab^	6.85^Aa^	0.438	<0.001	<0.001	<0.001
60	8.64^Ba^	11.74^Bc^	16.58^Be^	13.07^d^	17.18^Bf^	12.95^Bd^	10.75^Bb^	8.92^Bb^				
Acetic acid (g/kg DM)												
30	0.39^Bb^	0.52^Bc^	0.69^Be^	0.62^Bd^	0.02^a^	0.02^Aa^	0.42^Bb^	0.39^Ab^	0.030	0.091	0.271	0.002
60	0.17^Ac^	0.26^Ad^	0.33^Af^	0.34^Af^	0.01^a^	0.14^Bb^	0.29^Ae^	0.51^Bg^				
Propionic acid (g/kg DM)												
30	2.07^Ac^	1.67^a^	1.82^Ab^	2.32^Ad^	2.33^Bd^	2.09^Ac^	1.82^Ab^	2.57^Ae^	0.075	<0.001	<0.001	<0.001
60	2.17^Bc^	1.61^a^	2.11^Bc^	2.48^Be^	1.77^Ab^	2.25^Bd^	3.10^Bf^	3.64^Bg^				
Butyric acid (g/kg DM)												
30	0.38^Ad^	0.19^c^	0.39^Bd^	0.44^Be^	0.04^a^	0.03^b^	0.12^Ab^	0.37^Bd^	0.022	<0.001	<0.001	<0.001
60	0.50^Be^	0.16^b^	0.21^Ac^	0.22^Ac^	0.03^a^	0.01^a^	0.30^Bd^	0.29^Ad^				
Ammonia nitrogen/total nitrogen												
30	40.8^c^	31.3^Ab^	30.0^Ab^	27.3^Ab^	3.4^Aa^	3.4^Aa^	39.8^Ac^	38.2^Ac^	2.811	<0.001	<0.001	<0.001
60	43.9^c^	38.5^Bb^	39.5^Bb^	36.7^Bb^	7.1^Ba^	6.2^Ba^	64.1^Bd^	70.2^Be^				

CK, distilled water; LP, *L. plantarum*; LB, *L. buchneri*; LP + LB, *L. plantarum* and *L. buchneri*; PA, propionic acid; LP + PA, *L. plantarum* and propionic acid; CAP, calcium propionate; LP + CAP, *L. plantarum* and calcium propionate. D = ensilage days effect; T = treatment effect; D × T = the interaction between ensilage days and treatment. Means of additive treatment within a row followed by different lowercase superscripts differ from each other (*p* < 0.05). Means of ensiling time within a column followed by different uppercase superscripts differ from each other (*p* < 0.05).

After 30 days of ensiling, the LB and LP + LB treatments increased acetic acid content, and after 60 days of ensiling all treatments had greater (*p* < 0.05) lactic acid content than CK. The LP treatment had the lowest pH and highest lactic acid content of all treatments. It is likely that lactic acid has a lower pH than other organic acids and plays a vital role during fermentation (Jaipolsaen et al., 2021). In this study, the concentration of lactic acid at 30 days was greater (*p* < 0.05) than at 60 days of ensiling in all treatments, except for the PA treatment. Concomitantly, there was a decrease in acetic acid content, which implied that homolatic fermentation dominated. Li et al. (2018) concluded that the addition of *L. plantarum* enhanced the quality of alfalfa silage. At 30 days of ensiling, the LP + LB, PA, and LP + CAP treatments had greater (*p* < 0.05), but the LP, LB and CAP treatments (*p* < 0.05) had lesser concentrations of propionic acid than CK. The increase in propionic acid content was likely a result of lactic acid being consumed by *L. buchneri* (Li et al., 2018). With the prolongation of the ensiling period, the content of propionic acid in the CAP treatment increased (*p* < 0.05), which could be attributed to the dissociation of corresponding organic acid salts (Dai et al., 2022), as was reported by Wen et al. (2017). Kung et al. (2018) reported that butyric acid below 5 g/kg DM was optimal for high quality fermentation, and the butyric acid content of all treatments in the present study met this criterium. After an increase in the ensiling period, the content of butyric acid was lesser (*p* < 0.05) in the LP, PA, LP + PA and CAP treatments than in CK. The production of silage undergoes dynamic enzymatic and microbial processes of which the degradation of proteins is one of the most crucial stages (Wang S. et al., 2022). In the present study, the NH_3_-N:TN ratios in the PA and LP + PA treatments were lesser (*p* < 0.05) than in the other treatments, probably because the lower pH inhibited the activity of the protease (Tian H. et al., 2022). In addition, the NH_3_-N:TN ratios in the LP, LB and LP + LB treatments were lesser (*p* < 0.05) than in CK. *L. plantarum* inhibits protein degradation through its effect on enzymes and microorganisms (Xian et al., 2022), whereas, *L. buchneri*, through its bacteriostatic effect, reduces the degradation of protein by undesirable microorganisms. The combination of *L. buchneri* and *L. plantarum* had a synergistic effect in reducing the degradation of protein.

### 3.3. Correlations and principal component analysis of silage indices of hybrid *Pennisetum*

**Figure 1:**
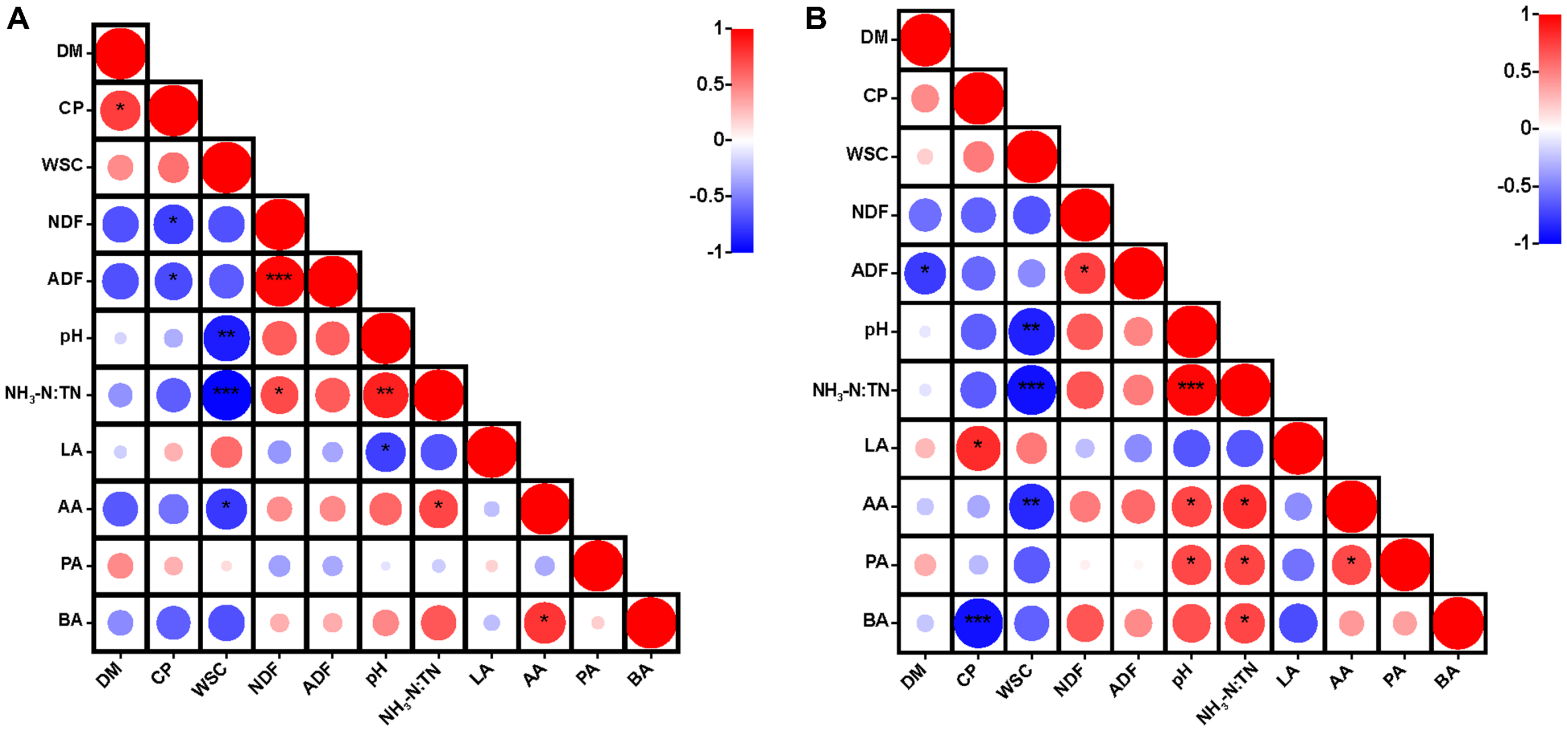
Heatmap of Pearson correlations between nutritional quality and fermentation parameters after 30 days **(A)** and 60 days **(B)** of ensiling hybrid *Pennisetum*. *p < 0.05, **p < 0.01, ***p < 0.001. DM, dry matter; CP, crude protein; WSC, water-soluble carbohydrates; NDF, neutral detergent fiber; ADF, acid detergent fiber; NH_3_-N:TN, ammonia nitrogen:total nitrogen ratio; LA, lactic acid; AA, acetic acid; PA, propionic acid; BA, butyric acid.

After 30 days of ensiling, CP was correlated positively (*p* < 0.05) with DM content, but negatively (*p* < 0.05) with NDF and ADF contents (Figure 1A). After 60 days of ensiling, CP content was correlated positively (*p* < 0.05) with lactic acid content and negatively (*p* < 0.001) with butyric acid content (Figure 1B). The WSC content was correlated negatively with acetic acid content (*p* < 0.05), pH (*p* < 0.01) and the NH_3_-N:TN ratio (*p* < 0.001). Moreover, the WSC content was correlated negatively with pH as a result of the lactic acid produced by LAB, with WSC as a substrate (Filya, 2003). Lactic acid plays the major role in reducing the pH of silage (Fu et al., 2022). After 30 days of ensiling, there was a positive correlation (*p* < 0.05) between pH and the NH_3_-N:TN ratio, and this correlation was stronger after 60 days of ensiling. Generally, NH_3_-N accumulates continuously during fermentation (Dong et al., 2022a). When LAB dominated in the late stages of ensiling, lactic acid was produced by the fermentation of plant biomass and the pH was reduced to a level that inhibited the activity of ammonia nitrogen producing bacteria (Fan et al., 2022). This could explain the correlation between pH and the NH_3_-N:TN ratio.

Principal component analysis (PCA) not only reduces the loss of original information, but also simplifies multiple related indicators into independent components, and, subsequently, assesses the indicators based on the difference in principal component scores (Gallo et al., 2013). In the present study, the PCA of 11 indicators of hybrid *Pennisetum* silage ensiled for different time lengths was carried out. The results of the PCA after 30 days of ensiling are presented in Tables 3, 4. The cumulative contribution of three extracted principal components, based on the characteristic value >1, reached 86.7%, that is, 86.7% of the original index was retained. The positive load value of the NH_3_-N:TN ratio and the negative load value of WSC were the greatest in the first principal component in their corresponding eigenvector, indicating that the WSC content could limit the silage quality of hybrid *Pennisetum*. In the second principal component, the positive load value of DM content and the negative load value of lactic acid were the greatest, indicating that the content of lactic acid was the chief factor limiting the quality of hybrid *Pennisetum* silage. The butyric acid content positive load value and the NDF negative load value were the greatest in the third principal component, which meant that the NDF content held a dominant position in limiting the quality of hybrid *Pennisetum* silage. The first principal component was correlated positively with NH_3_-N:TN, NDF, ADF and pH, and negatively with CP and WSC. The second principal component was correlated positively with DM, pH and propionic acid, and negatively with lactic acid and WSC, and the third principal component was correlated positively with organic acids such as butyric acid. After 60 days of ensiling, the cumulative contribution rate of the three extracted principal components, based on the characteristic value >1, reached 89.7% (Tables 3, 4). Similar to ensiling for 30 days, WSC, ADF and butyric acid were the main limiting factors of the principal components after ensiling for 60 days.

**Table 3:** Index coefficient and contribution rate of hybrid *Pennisetum* silage.

Item	Principal component (30d)			Principal component (60d)		
	PC1	PC2	PC3	PC1	PC2	PC3
Dry matter	−0.264	0.526	−0.164	−0.143	0.577	−0.136
Crude protein	−0.313	0.267	−0.092	−0.314	0.197	0.476
Water-soluble carbohydrates	−0.365	−0.209	−0.078	−0.345	−0.150	−0.243
Neutral detergent fiber	0.338	−0.175	−0.279	0.294	−0.302	0.235
Acid detergent fiber	0.328	−0.192	−0.245	0.268	−0.399	0.223
pH	0.319	0.370	−0.154	0.357	0.181	0.043
NH_3_-N:TN	0.369	0.213	0.003	0.370	0.178	0.085
Lactic acid	−0.218	−0.464	0.357	−0.294	−0.035	0.468
Acetic acid	0.318	−0.039	0.323	0.308	0.155	0.441
Propionic acid	−0.132	0.359	0.467	0.236	0.511	0.013
Butyric acid	0.271	0.112	0.589	0.319	−0.064	−0.410
Eigenvalue	6.375	1.823	1.339	6.618	2.105	1.145
Variance contribution rate (%)	57.95	16.57	12.17	60.16	19.14	10.41
Cumulative contribution rate (%)	57.95	74.53	86.70	60.16	79.30	89.71

NH_3_-N:TN, Ammonia nitrogen/total nitrogen.

**Table 4:** Principal component score, comprehensive score and ranking of hybrid *Pennisetum* silage.

Days	Treatment	F1	F2	F3	F	Ranking
30	LP + PA	−4.70	0.69	−0.50	−3.08	1
	PA	−2.85	−1.40	−0.27	−2.21	2
	LP + LB	−0.25	−0.33	2.44	0.10	3
	LP	1.03	−1.25	−0.56	0.37	4
	CAP	1.44	0.68	−1.61	0.87	5
	LP + CAP	0.75	2.75	0.44	1.08	6
	LB	2.06	−0.57	0.10	1.28	7
	CK	2.53	−0.57	−0.05	1.58	8
60	PA	−4.50	0.85	−0.26	−2.57	1
	LP + PA	−3.01	−0.07	−0.36	−1.86	2
	LP + LB	−0.23	0.61	0.33	0.01	3
	LP	0.63	−2.22	0.68	0.03	4
	LB	0.31	−1.07	1.58	0.15	5
	CK	1.85	−1.42	−2.12	0.62	6
	CAP	1.83	1.74	−0.27	1.41	7
	LP + CAP	3.11	1.58	0.42	2.22	8

F1, score of the first principal component; F2, score of the second principal component; F3, score of the third principal component; F, comprehensive score of principal components; CK, distilled water; LP, *L. plantarum*; LB, *L. buchneri*; LP + LB, *L. plantarum* and *L. buchneri*; PA, propionic acid; LP + PA, *L. plantarum* and propionic acid; CAP, calcium propionate; LP + CAP, *L. plantarum* and calcium propionate.

We concluded that the lower composite scores indicated better silage quality according to the composite of each original index and the proportion of principal components. Therefore, the top three treatments were LP + PA, PA, and LP + LB.

### 3.4. pH and microbial abundances of hybrid *Pennisetum* after aerobic exposure

The changes in pH and relative abundances of microorganisms during aerobic exposure after 60 days of ensiling are presented in Table 5. The resistance against spoilage varies greatly among silages, and different additives are used to prevent aerobic spoilage (Puntillo et al., 2020; Ferrero et al., 2021). With an increase in aerobic exposure, the pH increased (*p* < 0.05) in all treatments, except for the PA and LP + PA treatments. When the pH of the silage increases by 0.5 after aerobic exposure, it could be regarded as aerobic deterioration (Mu et al., 2021). In the current study, the pH of only the PA and LP + PA treatments did not increase by 0.5 after 6 days of aerobic exposure. The pH of the CAP and LP + CAP treatments were always greater (*p* < 0.05) than that of CK during aerobic exposure. During aerobic exposure, the abundance of LAB in the CK, LP, LB and LP + LB treatments displayed an increasing trend between days 0 and 6 and then a decreasing trend between days 6 and 9 (*p* < 0.05), while the abundance of LAB in the PA, LP + PA, CAP and LP + CAP treatments displayed an increasing trend (*p* < 0.05). When the number of yeasts exceeded 5 log_10_ cfu/g FW, the silage was prone to aerobic spoilage (Chen et al., 2016). In this study, the number of yeasts in each treatment, except for the organic acid treatment, exceeded this number at 6 days of aerobic exposure, indicating a trend of aerobic spoilage (Wang et al., 2014). The activity of aerobic microorganisms increased after the silage was exposed to air, and they used lactic acid, sugars and amino acids to produce heat continuously, resulting in aerobic putrefaction (Hu et al., 2009; Zhou et al., 2019). In this study, PA and LP + PA inhibited the proliferation of aerobic microorganisms, while the abundance of aerobic bacteria in the other treatments exhibited an increasing trend. The counts of yeast and aerobic bacteria in the PA treatment during aerobic exposure were lower (*p* < 0.05) than in other treatments, while the pH remained stable, and was lower (*p* < 0.05) than in other treatments on day 9, which improved the aerobic stability of the silage. The LB treatment failed to inhibit the proliferation of yeast, resulting in an increase (*p* < 0.05) in pH during aerobic exposure. It is likely that the undesirable microorganisms used the high content of residual lactic acid as a substrate after aerobic exposure (Rabelo et al., 2019).

**Table 5:** Changes of pH and microbial quantity of hybrid *Pennisetum* silage during aerobic exposure.

Item and ensiling days	Treatments								SEM		*P*	
	CK	LP	LB	LP + LB	PA	LP + PA	CAP	LP + CAP		D	T	D × T
pH												
0	4.30^Af^	4.18^Ad^	4.16^Ac^	4.28^Ae^	3.48^Ab^	3.44^Aa^	4.86^Ag^	4.89^Ah^	0.176	<0.001	<0.001	<0.001
3	5.12^Bd^	5.59^Be^	4.86^Bc^	4.66^Bb^	3.91^Ca^	3.89^Ca^	6.19^Bg^	5.87^Bf^				
6	7.14^Cd^	7.71^Cf^	7.26^Ce^	7.04^Cc^	3.86^Bb^	3.81^Ba^	8.16^Ch^	7.93^Cg^				
9	7.75^Dc^	7.88^Dd^	7.87^Dd^	8.09^De^	4.67^Da^	6.21^Db^	8.67^Dg^	8.44^Df^				
Lactic acid bacteria (log10 cfu/g FW)												
0	2.12^Aa^	3.63^Ac^	3.18^Ab^	3.74^Ac^	2.19^Aa^	2.20^Aa^	4.64^Ad^	4.59^Ad^	0.192	<0.001	<0.001	<0.001
3	2.36^Ba^	5.67^Bf^	3.79^Bc^	3.98^Ad^	2.88^Bb^	2.40^Aa^	5.06^Be^	5.21^Be^				
6	7.00^Cc^	7.06^Ccd^	7.53^Dg^	7.38^Cf^	3.21^Ca^	7.08^Bd^	6.70^Cb^	7.18^Ce^				
9	6.22^Dc^	5.90^Bb^	6.68^Cd^	6.77^Bd^	3.78^Da^	7.08^Be^	6.71^Cd^	7.46^Df^				
Yeast (log10 cfu/g FW)												
0	3.42^Ad^	4.16^Ae^	3.28^Ad^	3.41^Bd^	0.11^Aa^	0.52^Ab^	2.49^Ac^	4.48^Af^	0.223	<0.001	<0.001	<0.001
3	3.86^Bc^	5.77^Be^	3.62^Ac^	2.63^Ab^	2.36^Ba^	2.68^Bb^	5.09^Bd^	5.03^Bd^				
6	7.16^Cc^	7.25^Ccd^	7.45_Be_	7.31^Dd^	3.04^Ca^	2.93^Ba^	6.37^Cb^	7.28^Ccd^				
9	8.10^Df^	8.31^Df^	7.18^Be^	6.23^Cc^	4.52^Da^	4.98^Cb^	6.77^Dd^	7.16^Ce^				
Aerobic bacteria (log_10_ cfu/g FW)												
0	4.31^Ac^	4.24^Ac^	3.77^Ab^	4.18^Bc^	2.26^Aa^	2.09^Aa^	4.46^Acd^	4.60_Ad_	0.204	<0.001	<0.001	<0.001
3	4.97^Be^	5.76^Bf^	3.81^Ab^	3.77^Ab^	4.79^Bd^	3.19^Ba^	4.35^Ac^	4.74^Bd^				
6	7.33^Ccd^	7.66^Ce^	7.37^Bd^	7.24^Cc^	5.79^Cb^	3.52^Ca^	7.62^Be^	7.89^Cf^				
9	8.86^Dd^	8.85^Dd^	8.31^Cc^	8.33^Dc^	5.84^Cb^	5.70^Da^	8.26^Cc^	8.35^Dc^				

CK, distilled water; LP, *L. plantarum*; LB, *L. buchneri*; LP + LB, *L. plantarum* and *L. buchneri*; PA, propionic acid; LP + PA, *L. plantarum* and propionic acid; CAP, calcium propionate; LP + CAP, *L. plantarum* and calcium propionate. D = ensilage days effect; T = treatment effect; D × T = the interaction between ensilage days and treatment. Means of additive treatment within a row followed by different lowercase superscripts differ from each other (*p* < 0.05). Means of ensiling time within a column followed by different uppercase superscripts differ from each other (*p* < 0.05).

### 3.5. Microbial diversity

**Figure 2:**
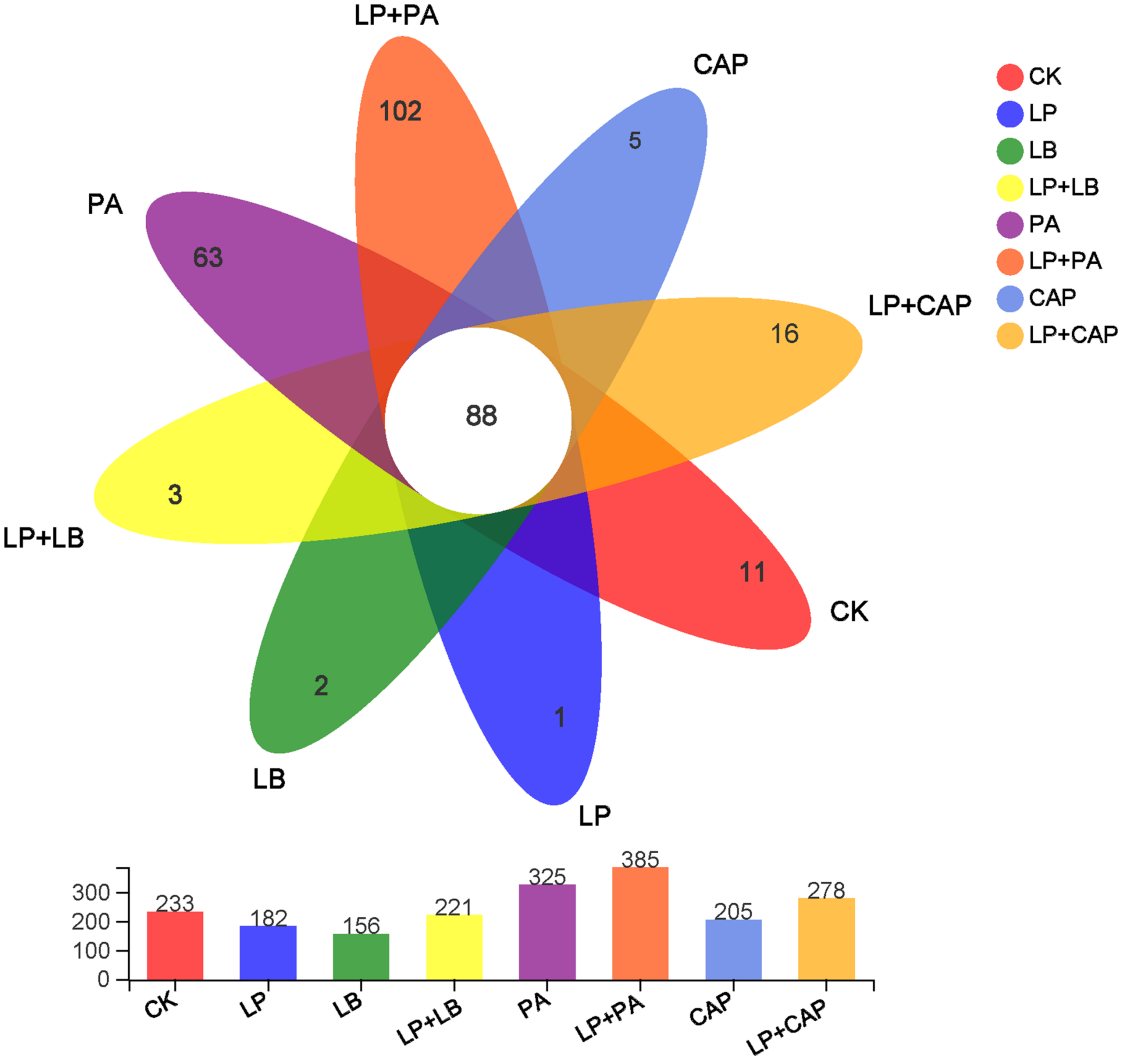
Petal diagram illustrating the degree of overlap of bacterial operational taxonomic units (OTUs) in the 8 silages. CK, distilled water; LP, *L. plantarum*; LB, *L. buchneri*; LP + LB, *L. plantarum* and *L. buchneri*; PA, propionic acid; LP + PA, *L. plantarum* and propionic acid; CAP, calcium propionate; LP + CAP, *L. plantarum* and calcium propionate.

**Figure 3:**
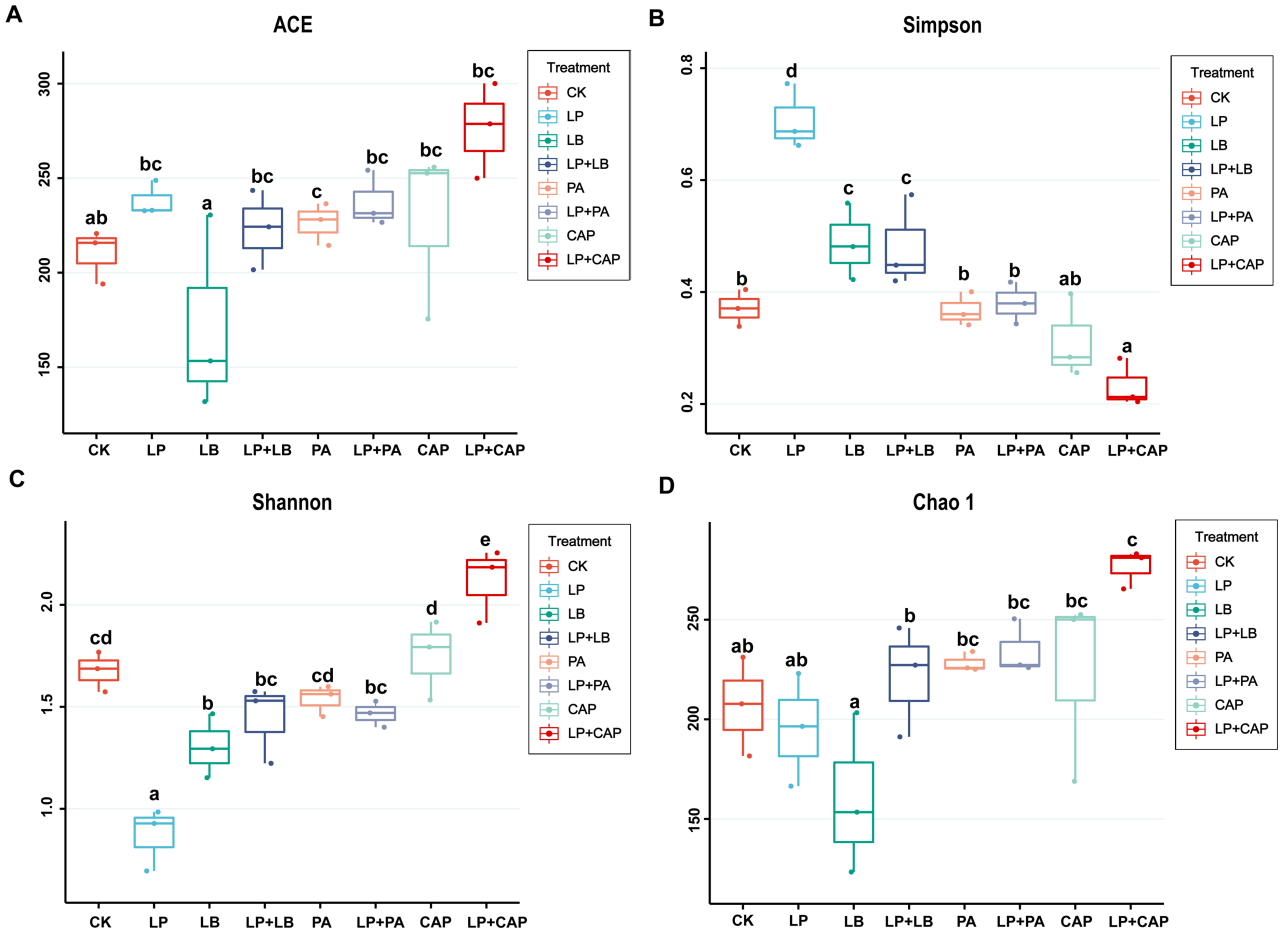
Effects of different additives on alpha diversity of hybrid *Pennisetum* silage. ACE **(A)**, Simpson **(B)**, Shannon **(C)** and Chao1 **(D)** indexes are used to reflected alpha diversity. CK, distilled water; LP, *L. plantarum*; LB, *L. buchneri*; LP + LB, *L. plantarum* and *L. buchneri*; PA, propionic acid; LP + PA, *L. plantarum* and propionic acid; CAP, calcium propionate; LP + CAP, *L. plantarum* and calcium propionate. Means with different lowercase letters differ from each other (*p* < 0.05).

After sequencing and quality control, a total of 1,044,312 optimized sequences were obtained. According to the 3% difference, a total of 1985 OTUs were obtained by OTU clustering. In total, 88 OTUs were shared by 8 processing units, accounting for 4.43% of all OTUs (Figure 2). CK, LP, LB, LP + LB, PA, LP + PA, CAP and LP + CAP had 11, 1, 2, 3, 63, 102, 5 and 16 OTUs, respectively. Alpha-diversity reflects the microbial abundance and species diversity of a single sample (Xian et al., 2022). Chao1 and ACE diversities are commonly used to measure species richness, while Shannon and Simpson indices are used to measure species diversity (Dong et al., 2020). Compared to CK, except for the LB treatment, the ACE index of all treatments increased, with the LP + CAP treatment being higher than the other treatments (Figure 3). The microbial community within the crop formed in the field, and when the stable environment was disrupted, the microorganisms that were not adapted to the fermentation system were eliminated, and the more adaptable microorganisms dominated in the new environment (Dong et al., 2022b). In this study, the Simpson index of LP was greater (*p* < 0.05) than the other treatments, while the Chao 1 index was greater (*p* < 0.05) and the ACE were lesser (*p* < 0.05) in the LP treatment than CK. Compared to CK, Shannon indices of LP and LB and the Simpson index of LP + CAP were lesser (*p* < 0.05), while the Shannon index of LP + CAP and the Simpson indices of LP, LB and LP + LB were greater (*p* < 0.05) than CK. The Shannon indices of LP + LB, LP + PA and LP + CAP were greater but the indices were lesser (*p* < 0.05) than the LP treatment (*p* < 0.05). The change in alpha diversity among silages was caused by the dynamic response of microorganisms (Feng et al., 2022). The composition and function of bacteria could differ during the period of ensiling (Sepehri and Sarrafzadeh, 2019).

**Figure 4:**
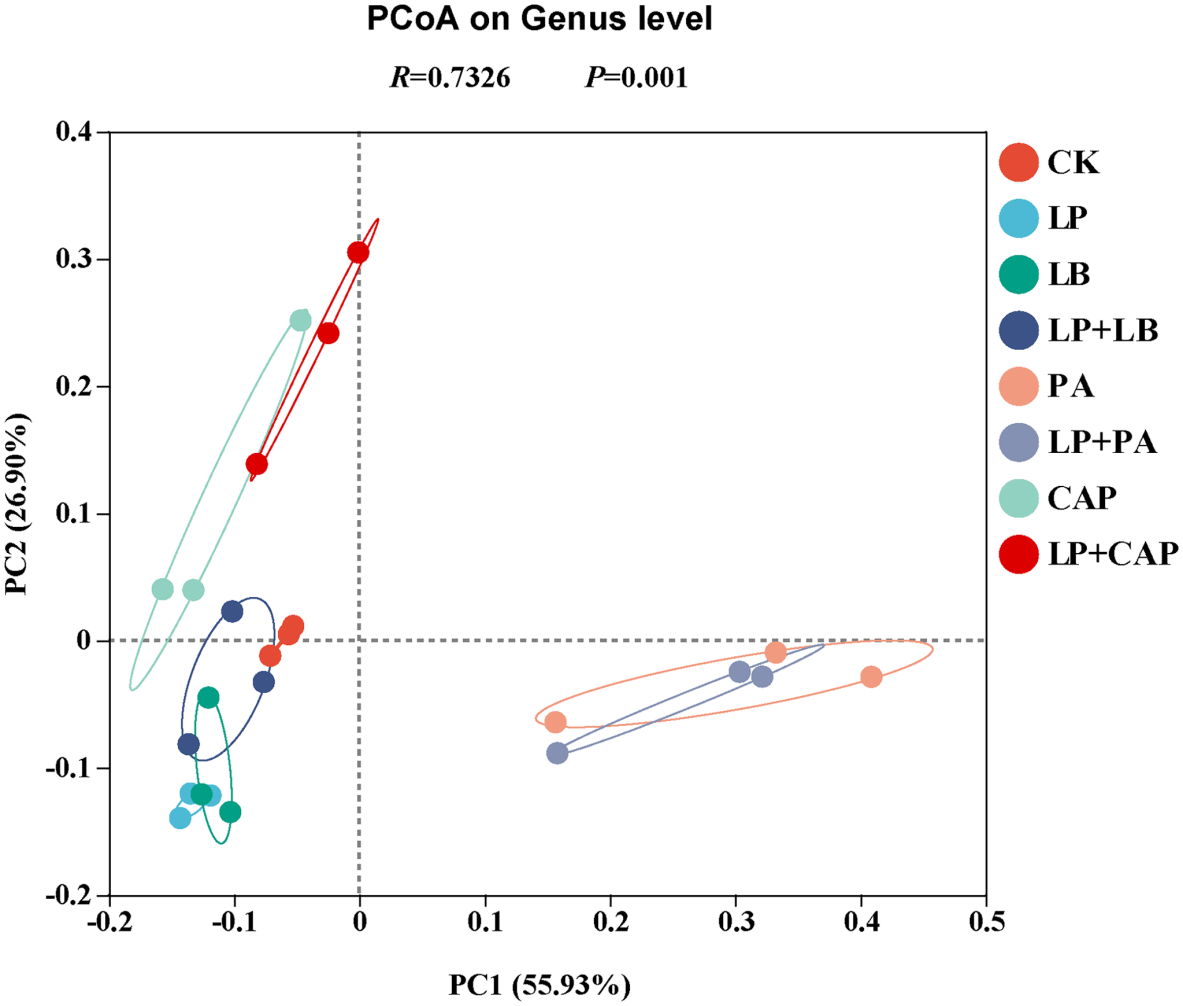
Principal coordinate analysis (PCoA) of microbial diversity of hybrid *Pennisetum* silage treated with different additives. CK, distilled water; LP, *L. plantarum*; LB, *L. buchneri*; LP + LB, *L. plantarum* and *L. buchneri*; PA, propionic acid; LP + PA, *L. plantarum* and propionic acid; CAP, calcium propionate; LP + CAP, *L. plantarum* and calcium propionate.

The PCoA based on the Bray–Curtis dissimilarity displayed distinct clusters among the eight silages (Figure 4). Further analysis through ANOSIM revealed that the results were reliable (*R* = 0.73, *p* = 0.001). According to PCoA, CK and LP + LB were clustered in the second and third quadrants, LP and LB in the third quadrant, PA and LP + PA mainly in the fourth quadrant, and CAP and LP + CAP mainly in the second quadrant. In addition, PA and LP + PA were separated from the other treatments, indicating that there were differences in the composition of species; whereas, CK was relatively close to LP, LB, and LP + LB, indicating that the composition of species was similar among these treatments. These results demonstrated that different additive treatments had significant effects on the bacterial community of hybrid *Pennisetum* silage (Tian J. et al., 2022).

**Figure 5:**
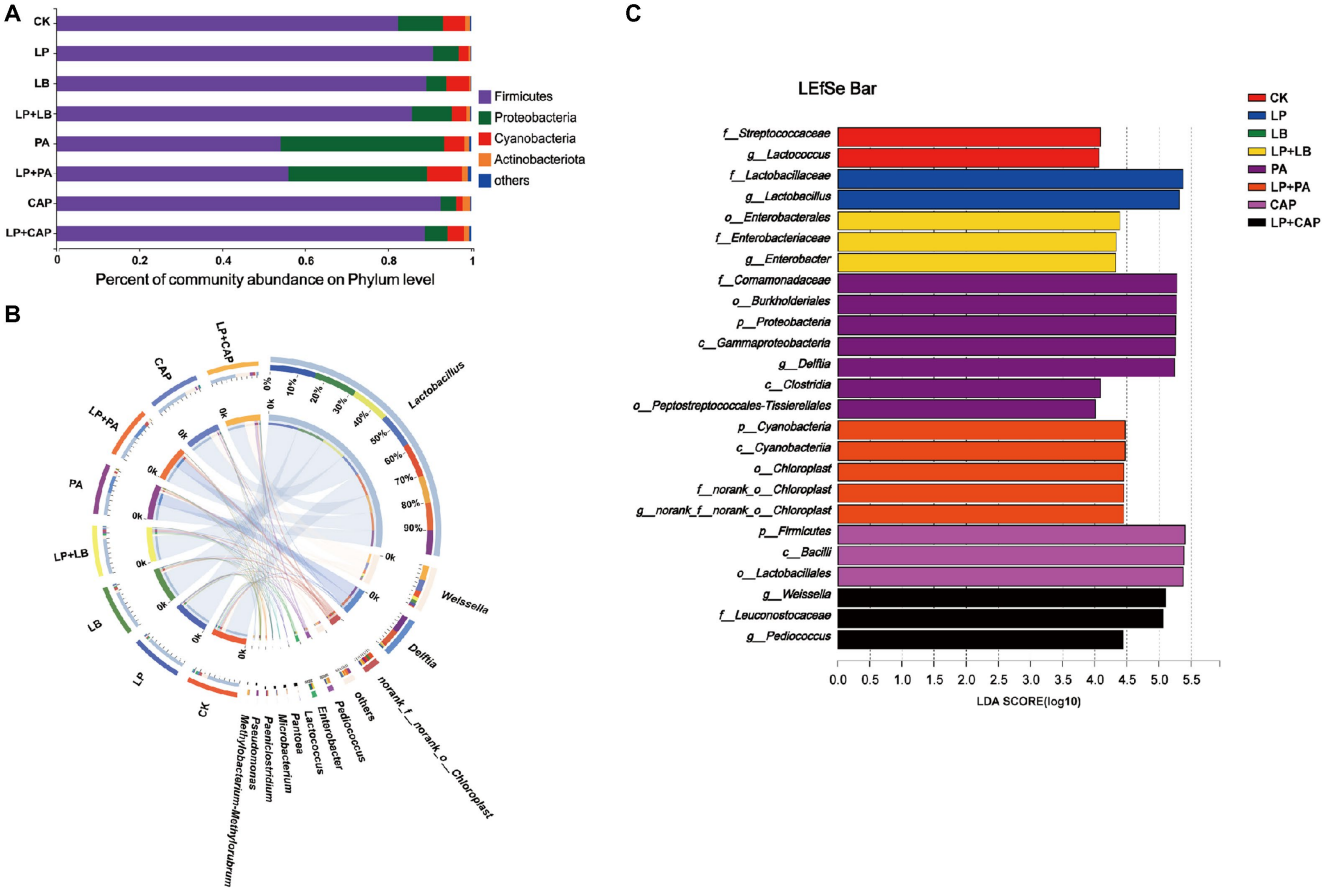
Bacterial abundance at phylum **(A)** and genus **(B)** levels of hybrid *Pennisetum* silage. Linear discriminant analysis effect size (LEfSe) of hybrid *Pennisetum* silage treated with different additives **(C)**. CK, distilled water; LP, *L. plantarum*; LB, *L. buchneri*; LP + LB, *L. plantarum* and *L. buchneri*; PA, propionic acid; LP + PA, *L. plantarum* and propionic acid; CAP, calcium propionate; LP + CAP, *L. plantarum* and calcium propionate.

After 60 days of ensiling hybrid *Pennisetum*, the main bacterial phylum was Firmicutes, followed by Proteobacteria and Cyanobacteria (Figure 5A). Liu et al. (2019) reported that Firmicutes and Proteobacteria were the most abundant phyla of barley silage at any point during the ensiling process with or without LAB inoculants, and continued to rise to 99% of the total bacteria at 60 days of ensiling. Most bacteria involved in lactic acid fermentation belong to Firmicutes and Proteobacteria and they play important roles in an anaerobic environment (Yuan et al., 2020). The relative abundance of Firmicutes was lesser (*p* < 0.05) and of Proteobacteria was greater (*p* < 0.05) in PA and LP + PA than in CK, while there was no difference (*p* > 0.05) in the other treatments. The relative abundance of Cyanobacteria in the LP and CAP treatments was lesser (*p* < 0.05), and in the LP + PA treatment was greater (*p* < 0.05) than in CK. Cyanobacteria is often found in tropical herbage and could be replaced by *Lactobacillus* and *Enterobacteria* after fermentation, but further studies are needed to determine their roles in silage production (Li et al., 2019). The relative abundance of Actinobacteriota in LB was lesser (*p* < 0.05), while of Actinobacteriota in CAP and LP + CAP was greater (*p* < 0.05) than in CK. When mixed with *L. plantarum*, the relative abundances of Firmicutes and Cyanobacteria in LP + PA were greater (*p* < 0.05), while the relative abundance of Proteobacteria was lesser (*p* < 0.05) than in LP. The relative abundance of Actinobacteriota in the LP + PA and LP + CAP treatments was greater (*p* < 0.05) than in the LP treatment. It was reported that Actinobacteriota had the potential of bioremediation to degrade pesticides and heavy metals (Alvarez et al., 2017).

The bacterial genera after 60 days of ensiling hybrid *Pennisetum* is presented in Figure 5B. *Lactobacillus* was the dominant genus in each treatment. Xu et al. (2020) reported that *Lactobacillus* was often the most important microbe in the late stages of ensiling, which, together with *Weissella* and *Pediococcus*, were the main producers of lactic acid. The relative abundance of *Lactobacillus* in LP (85.4%) was greater (*p* < 0.05), but in PA (46.8%) was lesser (*p* < 0.05) than in CK. The relative abundance of *Weissella* in the LP, LB, PA and LP + PA treatments was lesser (*p* < 0.05) than in CK (12.7%). *Weissella* belongs to the heterofermentative bacteria and consumes WSC to produce a mixture of lactic acid and acetic acid (Huang et al., 2021; Sun et al., 2021). The relative abundance of *Delftia* in the PA and LP + PA treatments was greater (*p* < 0.05) than in CK. *Delftia* is often present in soil and plants and promotes the growth and bioremediation of plants (Liu et al., 2018; Bhat et al., 2022). Dong et al. (2022b) concluded that the low pH of the silage might be due to an accumulation of nitrite in the silage. This occurred because *Delftia*, as a reductant of nitrate, could not reduce nitrite further. In the acidic environment of silage, nitrite could be converted into nitrogen oxides to reduce the pH of silage. The addition of organic acids reduced the relative abundances of *Klebsiella*, *Paenibacillus*, and *Enterobacter* in corn silage (Jiang et al., 2020). In the present study, the relative abundances of *Enterobacter* in the PA and LP + PA treatments and of *Enterobacterin* in the LP + LB treatment were greater (*p* < 0.05) than in CK. Guo et al. (2020) reported that *Enterobacter* was one of the dominant bacteria during ensiling, especially in silage treated with LAB. However, *Enterobacter* was unwanted due to nutrient loss caused by acetic acid fermentation (Ni et al., 2017b), although most *Enterobacter* bacteria in silage were considered non-pathogenic (Santos et al., 2016). The relative abundances of *Weissella* and *Pediococcus* in LP + CAP were greater (*p* < 0.05) than in LP, while the relative abundances of *Delftia* and *norank_o__Chloroplast* in LP + PA were greater (*p* < 0.05), but the relative abundance of *Enterobacter* in LP + PA and LP + CAP was lesser (*p* < 0.05) than in CK. The relative abundance of *Methylobactere-methylorubrum* in LP + CAP (1.42%) was greater (*p* < 0.05) than in the other treatments. *Methylobactery-methylorubrum* is a gram negative, rod-shaped, strictly aerobic bacteria that can utilize methanol and other reduced one-carbon compounds via the serine pathway (Dong et al., 2022b). The high pH of the LP + CAP treatment after 60 days of ensiling was consistent with the neutrophilic property of *Methylobacter-methylorubrum* (Knief et al., 2012). *Methylobacterium* was the dominant genus in alfalfa silage (Ni et al., 2017a; Ogunade et al., 2018).

LEfSe was used to analyze the different bacteria species in each treatment. A total of 25 species with relative abundance differences was identified in the 8 treatments (LDA score > 4) (Figure 5C). *Proteobacteria* and *Delftia* were biomarkers of the PA treatment at the phylum and genus levels, respectively, while *Cyanobacteria* and *Enterobacter* were biomarkers of the LP + PA treatment. Firmicutes was the most abundant phylum in the CAP treatment. In addition, the highest abundances of each treatment at the genus level were as follows: *Lactococcus* in CK, *Lactobacillus* in LP, and *Weissella* and *Pediococcus* in LP + CAP. Previous studies reported that *Pediococcus* was generally highly abundant in silage with high pH (Zhao et al., 2022; Zong et al., 2022), and the treatment with LP + CAP in this study had the highest pH at 60 days of ensiling (Table 2).

**Figure 6:**
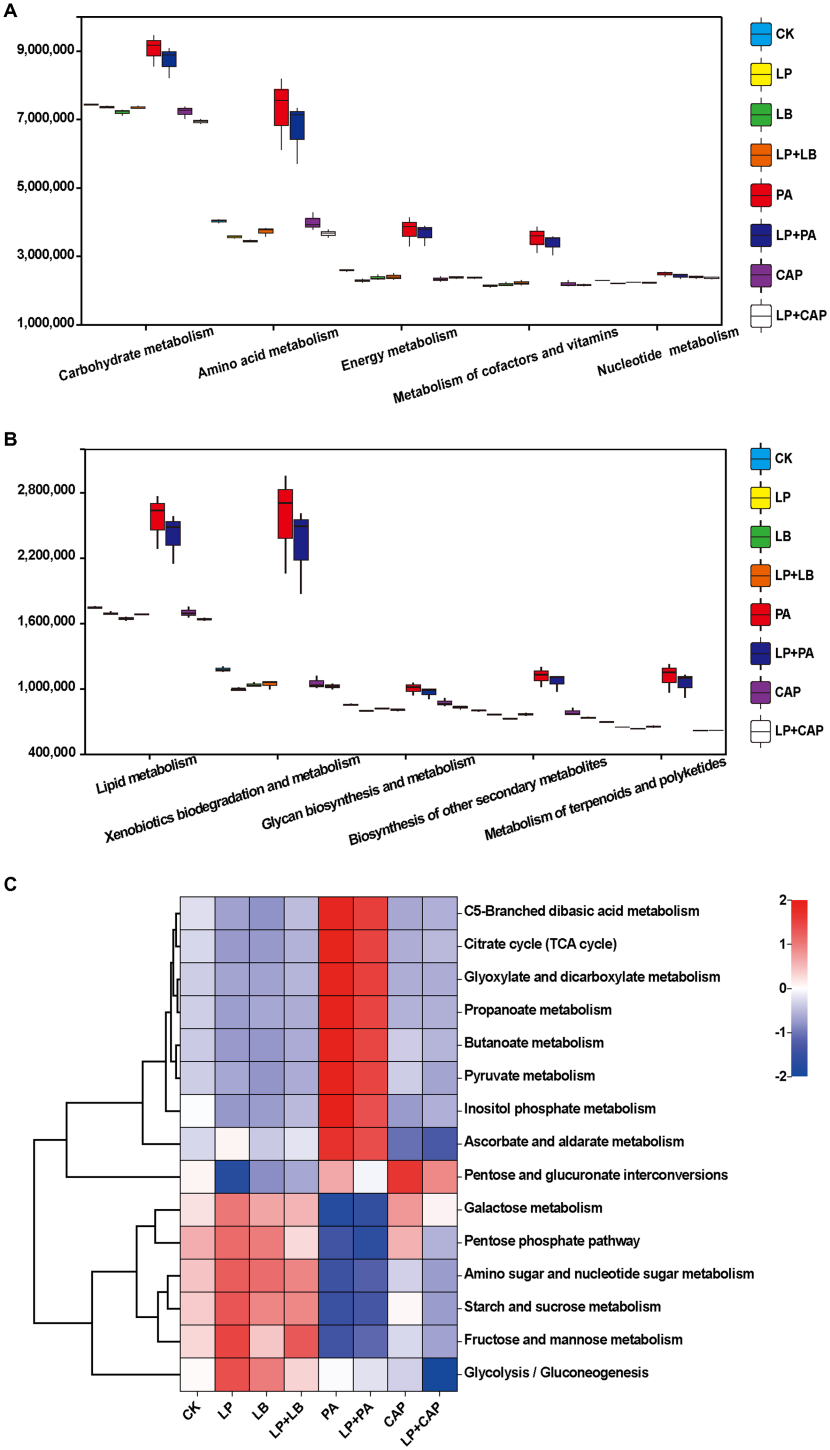
Prediction of microbial metabolic functions of hybrid *Pennisetum* silage. The second-level **(A,B)** and third-level **(C)** classification of microbial community metabolic functions. CK, distilled water; LP, *L. plantarum*; LB, *L. buchneri*; LP + LB, *L. plantarum* and *L. buchneri*; PA, propionic acid; LP + PA, *L. plantarum* and propionic acid; CAP, calcium propionate; LP + CAP, *L. plantarum* and calcium propionate.

Figures 6A,B present the second-level classification of microbial community metabolic functions. The metabolic pathway with the greatest abundance in all treatments was carbohydrate metabolism. This metabolism was greater in the PA and LP + PA treatments than in the other treatments, perhaps due to the their high WSC content (Table 1). In addition, the lactic acid content in the PA and LP + PA treatments was greater (*p* < 0.05) than in CK, which most likely was due to carbohydrate metabolism. The metabolism abundances of the CAP, LP + CAP, PA and LP + PA treatments were greater than CK (*p* < 0.05), which might be due to the response of microorganisms to long-term acid stress in silage (Bai et al., 2022). The metabolic abundances of terpenoids and polyketides were predicted to be relatively high in the PA and LP + PA treatments. Terpenoids are natural compounds, mainly in Chinese herbal medicine, and are reputed to possess antibacterial and antioxidant properties (Elshamy et al., 2016; Tian et al., 2017). Further predictions of the third-level metabolic pathways of carbohydrate metabolism are presented in Figure 6C. The PA and LP + PA treatments had similar metabolic functions in all carbohydrates, and both had greater metabolism of pyruvate, propanoate, butanoate, ascorbate and aldarate than CK. It was reported that pyruvate metabolism was related to the formation of organic acids such as lactic acid, α-acetolactic acid, acetic acid, and formic acid (Dong et al., 2022a). Pyruvate, an intermediate in the glycolytic pathway, is crucial in lactic acid generation by LAB utilizing WSC, and can interconvert sugars, fats, and amino acids through the acetyl CoA and tricarboxylic acid cycles. As mentioned above, the lactic acid contents of the PA and LP + PA treatments were greater than in CK, and the pH was the lowest among all treatments, which might be related to the up-regulation of this pathway. In addition, the increase in the metabolism of ascorbate and aldarate, C5-branched dibasic acid and inositol phosphate, and glyoxylate and dicarboxylate suggests the consumption of sugar (Yin et al., 2022).

**Figure 7:**
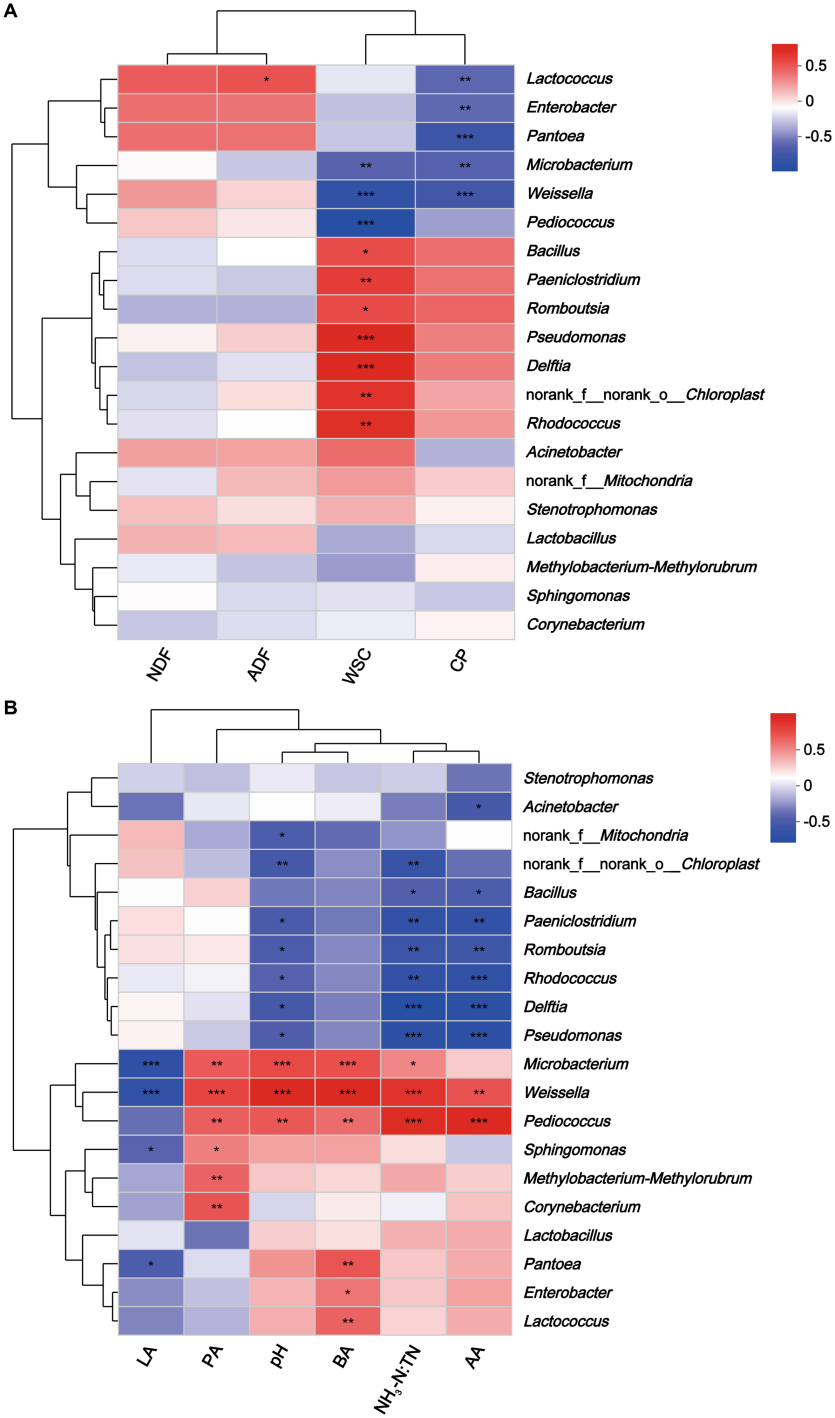
Heatmap of Spearman correlations between nutritional composition **(A)**, fermentation parameters **(B)** and bacterial abundance of hybrid *Pennisetum* silage. *p < 0.05; **p < 0.01, ***p < 0.001. NDF, neutral detergent fiber; ADF, acid detergent fiber; WSC, water-soluble carbohydrate; CP, crude protein; LA, lactic acid; AA, acidic acid; PA, propionic acid; BA, butyric acid; NH_3_-N:TN, ammonia nitrogen:total nitrogen ratio.

Spearman’s correlation tested the relationships between microbial communities at the genus level and nutrients and fermentation characteristics of hybrid *Pennisetum* silage. *Lactococcus* correlated positively with ADF (*r* = 0.44, *p* < 0.05), and negatively with CP (*r* = −0.53, *p* < 0.01) (Figure 7). Moreover, WSC correlated positively with *Pseudomonas* (*r* = 0.68, *p* < 0.001) and *Delftia* (*r* = 0.66, *p* < 0.001), and negatively with *Pediococcus* (*r* = −0.85, *p* < 0.001) and *Weissella* (*r* = −0.76, *p* < 0.001). WSC could be used by microorganisms as a substrate, so it was not surprising that WSC correlated negatively with microorganisms (Zheng et al., 2022). In addition, *Enterobacter* (*r* = −0.52, *p* < 0.01), *Pantoea* (*r* = −0.67, *p* < 0.001), and *Microbacterium* (*r* = −0.65, *p* < 0.001) were correlated negatively with CP. Previous studies had shown that certain *Enterobacter* were proteolytic, which could cause the loss of protein from silage (Yang et al., 2020). In this study, *Weissella* was correlated negatively with lactic acid content (*r* = −0.65, *p* < 0.001) and positively with acetic acid (*r* = 0.62, *p* < 0.01), propionic acid (*r* = 0.68, *p* < 0.001), butyric acid (*r* = 0.87, *p* < 0.001), pH (*r* = 0.87, *p* < 0.001) and NH_3_-N:TN (*r* = 0.76, *p* < 0.001). Results were consistent with those of Zheng et al. (2022) who reported that pH and the concentration of acetic acid were correlated positively with the abundance of *Weissella*. Furthermore, *Pediococcus* was correlated positively with NH_3_-N:TN (*r* = 0.83, *p* < 0.001) and acetic acid (*r* = 0.86, *p* < 0.001). Several studies indicated that *Pediococcus* possessed probiotics properties (Fugaban et al., 2021; Jiang et al., 2021). According to Yang et al. (2019), *Pediococcus* plays a major role in the initial stage of ensiling by helping to create an anaerobic environment that is suitable for LAB growth. *Microbacterium* might reduce silage quality as this bacterium correlated negatively with lactic acid and CP and positively with pH, butyric acid and NH_3_-N:TN. *Microbacterium* is a gram-positive bacterium belonging to Actinobacteria, and is generally isolated from terrestrial and aquatic ecosystems (Marchant et al., 2006). It was reported *Microbacterium* had the ability to degrade hydrocarbons and complex polysaccharides (Cordovez et al., 2018). However, its specific role in silage production warrants further research.

## 4. Conclusion

Additives affected the quality of hybrid *Pennisetum* silage by increasing crude protein and lactic acid contents and inhibiting the growth of undesirable bacteria. Principal component analysis revealed that the silage quality of the PA, LP + PA and LB + LP treatments ranked as the top three of the seven treatments. The synergistic effect of *L. plantarum* combined with *L. buchneri* improved the quality of silage more so than any one of them alone. The addition of propionic acid was very beneficial, as it increased the relative abundance of *Delftia*, inhibited the activity of *Enterobacter*, maintained pH, butyric acid and the NH_3_-N:TN ratio at low levels and reduced the contents of NDF and ADF. In summary, *L. plantarum*, *L. buchneri*, propionic acid, calcium propionate and their combinations could improve the silage of hybrid *Pennisetum*, which would mitigate the shortage of feed for livestock.

## Data availability statement

The datasets presented in this study can be found in online repositories. The names of the repository/repositories and accession number(s) can be found at: https://www.ncbi.nlm.nih.gov/, PRJNA946341.

## Author contributions

QF, JuZ, and WL: writing—original draft, formal analysis, and data curation. AD and CG: writing—review and editing. YZ: formal analysis and data curation. FY and JiZ: funding acquisition, supervision, and writing—review and editing. All authors contributed to the article and approved the submitted version.

## Funding

This work was supported by the National Natural Science Foundation of China (42075116 and 32101418), the Fujian Provincial Outstanding Youth Fund Projects (2023J01313545), and the Fujian Provincial Subsidy Project for Science and Technology Special Commissioner (2022S2071).

## Conflict of interest

The authors declare that the research was conducted in the absence of any commercial or financial relationships that could be construed as a potential conflict of interest.

## Publisher’s note

All claims expressed in this article are solely those of the authors and do not necessarily represent those of their affiliated organizations, or those of the publisher, the editors and the reviewers. Any product that may be evaluated in this article, or claim that may be made by its manufacturer, is not guaranteed or endorsed by the publisher.

## Footnotes

^1^ http://www.drive5.com/uparse/
